# A Patient-Specific CFD Pipeline Using Doppler Echocardiography for Application in Coarctation of the Aorta in a Limited Resource Clinical Context

**DOI:** 10.3389/fbioe.2020.00409

**Published:** 2020-06-03

**Authors:** Liam Swanson, Benjamin Owen, Amir Keshmiri, Amin Deyranlou, Thomas Aldersley, John Lawrenson, Paul Human, Rik De Decker, Barend Fourie, George Comitis, Mark E. Engel, Bernard Keavney, Liesl Zühlke, Malebogo Ngoepe, Alistair Revell

**Affiliations:** ^1^Department of Mechanical Engineering, University of Cape Town, Cape Town, South Africa; ^2^Department of Mechanical, Aerospace and Civil Engineering (MACE), The University of Manchester, Manchester, United Kingdom; ^3^Department of Paediatrics and Child Health, University of Cape Town, Cape Town, South Africa; ^4^Department of Paediatrics and Child Health, Tygerberg Hospital, Stellenbosch University and Tygerberg Hospital, Cape Town, South Africa; ^5^Christiaan Barnard Division of Cardiothoracic Surgery, University of Cape Town and Groote Schuur Hospital, Cape Town, South Africa; ^6^Department of Medicine, University of Cape Town, Cape Town, South Africa; ^7^Division of Cardiovascular Sciences, Faculty of Biology, Medicine and Health, School of Medical Sciences, The University of Manchester, Manchester, United Kingdom; ^8^Manchester University NHS Foundation Trust, Manchester Academic Health Science Centre, Manchester, United Kingdom

**Keywords:** coarctation of the aorta, computational fluid dynamics, Doppler echocardiography, patient-specific, congenital heart disease

## Abstract

Congenital heart disease (CHD) is the most common birth defect globally and coarctation of the aorta (CoA) is one of the commoner CHD conditions, affecting around 1/1800 live births. CoA is considered a CHD of critical severity. Unfortunately, the prognosis for a child born in a low and lower-middle income country (LLMICs) with CoA is far worse than in a high-income country. Reduced diagnostic and interventional capacities of specialists in these regions lead to delayed diagnosis and treatment, which in turn lead to more cases presenting at an advanced stage. Computational fluid dynamics (CFD) is an important tool in this context since it can provide additional diagnostic data in the form of hemodynamic parameters. It also provides an *in silico* framework, both to test potential procedures and to assess the risk of further complications arising post-repair. Although this concept is already in practice in high income countries, the clinical infrastructure in LLMICs can be sparse, and access to advanced imaging modalities such as phase contrast magnetic resonance imaging (PC-MRI) is limited, if not impossible. In this study, a pipeline was developed in conjunction with clinicians at the Red Cross War Memorial Children’s Hospital, Cape Town and was applied to perform a patient-specific CFD study of CoA. The pipeline uses data acquired from CT angiography and Doppler transthoracic echocardiography (both much more clinically available than MRI in LLMICs), while segmentation is conducted via SimVascular and simulation is realized using OpenFOAM. The reduction in cost through use of open-source software and the use of broadly available imaging modalities makes the methodology clinically feasible and repeatable within resource-constrained environments. The project identifies the key role of Doppler echocardiography, despite its disadvantages, as an intrinsic component of the pipeline if it is to be used routinely in LLMICs.

## Introduction

Congenital heart disease (CHD) has a global prevalence of near 9 per 1000 births (van der Linde et al., 2011; Liu et al., 2019) but the occurrence in Africa is reported to be significantly lower (Marijon et al., 2006; Mocumbi et al., 2011; Zühlke et al., 2019). Rather than being a reflection of geographical influences, this is attributed to a lack of data and reflects the restricted diagnostic and interventional freedom of specialists in under-resourced regions of the world (van der Linde et al., 2011; Zühlke et al., 2019). These restrictions lead to delayed treatment of patients who present with worsening severity and, consequently, a worse prognosis (Zühlke et al., 2019). Of CHDs, Coarctation of the aorta (CoA) is one of the commoner lesions, comprising ∼7% of all CHDs.

Coarctation of the aorta is defined by a stenosis at a point in the aorta, usually at the isthmus, which may be discrete or elongated, with varying degrees of severity. The consequent increase in resistance to flow causes upper body hypertension and reduced blood supply to the lower limbs. The pressure difference across the coarctation, clinically referred to as the pressure gradient, is commonly used to define the need for a repair as well as to gauge whether the repair was successful or not. Generally, a repair is recommended if the peak-systolic pressure difference is greater than 20-mmHg or when there is supporting imaging evidence of a coarctation with associated hypertension (Kumar and Clark, 2014; Nance et al., 2016). The common therapeutic techniques are balloon angioplasty, resection with end-to-end anastomosis (REEA) and, in older patients, stenting (Nance et al., 2016). Ultimately the goal is to alleviate the pressure difference and restore normal flow, but it is often found that hypertension can persist despite this being achieved, while atherosclerotic diseases and other morbidities develop later in life and recoarctation occurs (Cecchi et al., 2011; Kenny et al., 2011; Torok et al., 2015).

Indeed, the hemodynamic environment that exists *after* a repair is thought to inadvertently be a driver for the development of disease (Cecchi et al., 2011). Hence, research in patient-specific computational hemodynamics has been directed toward identifying a link between flow metrics such as pressure, velocity and wall shear stress (WSS), and the development of other cardiac lesions and recoarctation. Computational fluid dynamics (CFD) is seen as an important tool in this regard due to its high resolution of flow detail, by providing the capability to conduct *in silico* studies of different repairs and through incorporating predictive mathematical models for vascular growth and remodeling (Marsden and Feinstein, 2015; Capelli et al., 2018).

In low and lower-middle income countries (LLMICs), clinical infrastructure can be sparse and patient access to tertiary or specialist facilities is restricted (Rashid et al., 2016; Zühlke et al., 2019). This creates challenges to patient care in terms of follow up and procedure planning, and emphasizes the need for a deeper understanding of the driving causes for diseases which may develop post-repair. With greater insight, repair techniques may be optimized and patient follow-up care planned more effectively. CFD and patient-specific healthcare for CoA show synergies in this regard and there is great potential for a significant contribution toward patient management in resource-constrained countries.

In the context of a limited resource clinical setting in LLMICs, the design of a patient-specific CFD pipeline which specifically addresses clinical applicability requires careful consideration. The use of broadly available clinical infrastructure, together with capable and reliable open-source software packages is important so as to manage the cost of implementing such a tool. Current literature in patient-specific CFD favors the use of phase contrasted magnetic resonance imaging (PC-MRI) over Doppler transthoracic echocardiography (TTE) for the acquisition of velocity field data (Goubergrits et al., 2015; Doost et al., 2016). While the value of MRI as a data acquisition modality is not disputed, its availability in LLMICs is not broad enough to be relied upon as the modality for defining a CFD study. Advancement of CFD techniques in a clinical space will be crucial for the translation of the technology into clinical practice. While advanced imaging modalities contribute toward this end, this project investigates the feasibility of using Doppler echocardiography as a modality. Despite its potential disadvantages, the use of echo data in CFD modeling will be a requirement if less well-resourced nations are to benefit from computational methods.

In this study, a patient-specific CFD study of a single coarctation of the aorta patient is conducted based on a pipeline which was developed in conjunction with a team of clinicians at the Red Cross War Memorial Children’s Hospital (Red Cross). The premise of the pipeline design was to use open source software for clinical data processing and flow simulation and data that was acquired from CT Angiography (CTA) and Doppler echocardiography. Through this study, the importance of Doppler echocardiography is highlighted for its role in enabling access to patient-specific CFD simulations in LLMICs.

The implementation of open-source software and imaging modalities that are broadly available in LLMICs makes this methodology clinically feasible and repeatable within resource-constrained environments. The emphasis of the present work is to demonstrate the important role of Doppler echocardiography in LLMICs in providing patient-specific data for an open-source approach to data processing and numerical simulation. In the present work, commercial meshing software is used to provide a high-quality mesh, and while in the future we aim to use an opensource meshing tool instead, it was not felt to significantly alter the conclusion or the fundamental aim at this stage.

## Materials and Methods

At each point in the pipeline, from data collection through to CFD result post-processing, consideration had to be given to the clinical feasibility and repeatability of the method. The collaboration with the clinical team at Red Cross was thus important throughout the development such that focus remained on this goal.

### Patient Data Acquisition

A retrospective search of Red Cross CoA patient datasets found that the data collected through standard clinical protocol was not sufficient to define the patient-specific geometry and boundary conditions for a CFD simulation. To date, the standard clinical evaluation of a CoA performed at Red Cross uses several modalities and measurements as follows:

• Investigations typically begin with transthoracic echocardiography (with and without Doppler color mapping) to measure the continuation of flow through the stenosis during diastole (referred to as a diastolic tail), approximate the pressure difference across the stenosis and visualize and measure the stenosis.
• An x-ray angiogram may be conducted and a direct catheter-based peak-systolic pressure difference measurement taken across the stenosis.
• In some cases, a CTA may be conducted instead of an x-ray angiogram. It should be noted that cardiac MRI was not an available modality for CoA investigations.

This dataset is assessed as a whole to form the basis of the diagnosis and plan for treatment. In this process are evident limitations from both an infrastructural and safety perspective. A patient would not be exposed to both x-ray and CTA, due to the deleterious effects of the ionizing radiation; as such, in current clinical practice, it is not possible to have both a direct catheter pressure measurements and 3D volume visualization.

**FIGURE 1:**
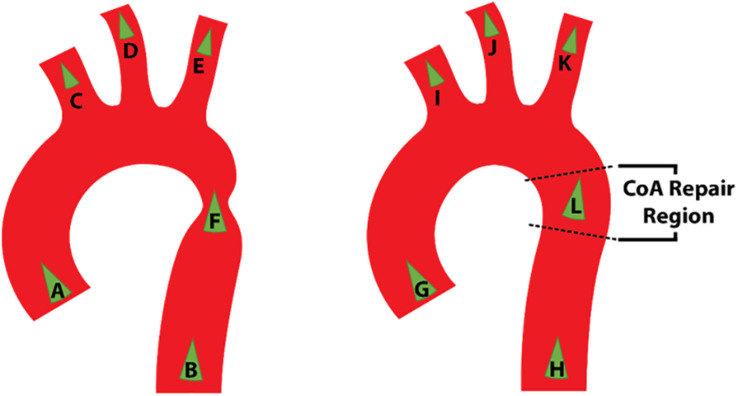
Schematic representation of the location of Doppler transthoracic echocardiographic measurement sites before (left) and after (right) intervention.

For the purpose of the present study, a CFD focused data collection protocol was developed in conjunction with the Red Cross clinical team so that the velocity, pressure and geometry of the CoA case could be defined at relevant locations. It was decided that imaging modalities for data collection would be restricted to CTA and Doppler echocardiography. It was important that the data collection be conducted within ethical bounds, and as such this protocol did not subject any patient to an investigation conducted beyond standard clinical care. However, at the discretion of the clinician and with the utmost regard for the patient, extra measurements were taken during a standard investigation for the purpose of this study. The acquisition of the Doppler measurements did not prolong the echo study by more than a few minutes and sedation was not used during echocardiographic studies. Doppler TTE was used to obtain flow data at the inlet, each outlet and at the site of the stenosis before and after the repair as shown in Figure 1. The Doppler TTE data was used to define the domain’s boundary conditions and give a point of reference for the maximum velocity and pressure difference at the coarctation to which the CFD results could be compared.

A patient admitted soon after the establishment of this data collection protocol was diagnosed with CoA at 18 months of age, for which CTA was conducted. This provided the opportunity to obtain pre-repair Doppler echocardiography velocity data that was necessary for the definition of the domain’s boundary conditions. At 19 months of age, following a repair, a Doppler echocardiography investigation, in line with the study’s protocol, was repeated in order to obtain post-repair velocity data necessary for the boundary conditions as well as geometry data in lieu of the CTA data. A CTA was not repeated after the repair due to concerns about unnecessary radiation exposure. The CTA and Doppler echocardiography dataset were anonymized and processed to be applied to their relevant CFD models.

### Geometry Segmentation and *in silico* Design

The 3D aorta geometry was manually segmented from the pre-repair CTA dataset using the open source SimVascular package (Updegrove et al., 2017). The region of interest (ROI) was chosen to extend from the aortic sinus to the descending aorta at the level of the diaphragm. It included the innominate artery (innominate), left common carotid artery (LCCA) and left subclavian artery (LSCA), collectively referred to as the supra-aortic branch vessels, up to the point prior to where the vessel bifurcated. The inlet, at the aortic sinus, was assumed to have a circular cross section in order to simplify the application of the inlet boundary condition.

**FIGURE 2:**
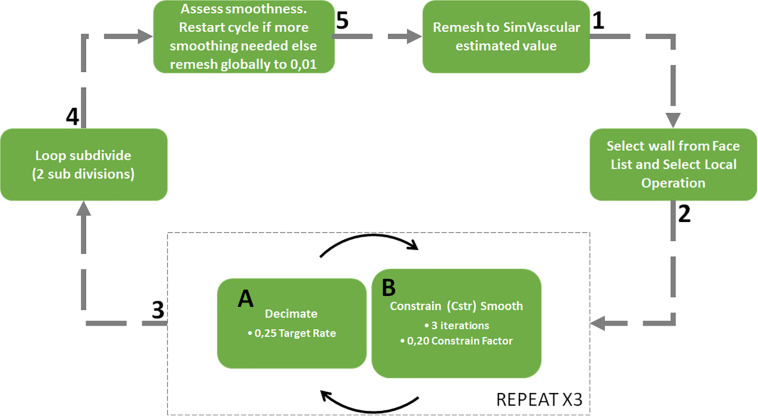
Surface meshing and corner smoothing cycle starting at 1 and following the arrows with sub cycle at phase 3.

The outlet patches were extruded by approximately ten times their hydraulic diameter to avoid intersection with a region of flow recirculation. Following this extension, all segmented vessels were merged into a PolyData surface which was smoothed in SimVascular using the process shown schematically in Figure 2.

Smoothing algorithms are important to smooth the sharp junction at bifurcation zones which may cause unrealistic flow features. The smoothing process illustrated in Figure 2 was weighted to control the deviation of the smoothed surface from the original vessel geometry yet still allow the sharp corners to be reduced. This was achieved through the constrain smoothing and decimation factors. The surface tends to become simplified after step three in the process and so two loops subdivision steps were carried out to smooth the geometry and increase the number of surface facets (Updegrove et al., 2016). Once the corners were sufficiently smooth, the surface was globally remeshed to a facet size of 0.01 mm to ensure that the surface detail would be sufficient for mesh generation.

In order to ensure accurate segmentation, the final surface geometry was superimposed onto the CTA DICOM data to check that the surface aligned with the illuminated blood in each slice. Furthermore, the geometry was assessed by the clinical team to confirm its fidelity and ensure that no other false anomaly had been introduced.

Since a post-repair CTA was not obtained, it was necessary to manually adjust the pre-repair geometry to form an approximation of the two further states tested; the approximate post-repair geometry and a hypothetical “healthy” state with the coarctation removed entirely. The geometries for these two cases were generated by expanding the segmentation contours in the diseased region of the pre-repair segmented geometry. The post-repair approximation was determined by matching the minimum diameter of the aorta at the site of the coarctation repair to the measurement in the post-repair Doppler echocardiography dataset. The hypothetical, healthy geometry was designed based on expanding the segmentation contours such that no constriction was present. The pre-repair, post-repair and healthy geometries are henceforth referred to as case 1, case 2, and case 3, respectively. All geometries were acknowledged by the clinical team as being reasonable representations.

### Doppler Echocardiography Data Processing

**FIGURE 3:**
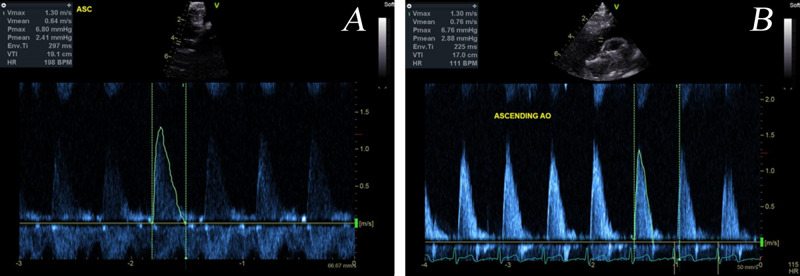
Example Doppler echocardiography dataset taken at the ascending aorta pre- **(A)** and post-repair **(B)**. The velocity time plot that was digitized is outlined.

The pre- and post-repair Doppler echocardiography investigations provided velocity data over several cardiac cycles at locations of interest in the domain. The Doppler echocardiography data that was collected at the ascending aorta is shown in Figure 3 as an example of the data that was obtained through such an investigation. The maximum indicated pressure difference was estimated using the simplified Bernoulli equation expressed in equation 1 which is derived by assuming that frictional losses and the proximal fluid velocity in the full Bernoulli equation are negligible (Heys et al., 2010). Furthermore, the co-efficient in the velocity term incorporates the density and a conversion factor from Pascal to the clinically preferable, mmHg units. The resulting, simplified Bernoulli equation is (Kumar and Clark, 2014):

(1) $$\Delta \,P=4\,v^{2}$$

The velocity data required processing so that it could be used for defining the boundary conditions. Firstly, the velocity-time plot over a cardiac cycle was digitized and smoothed. Since the heart rate of the patient varies during the measurements the period of each digitized plot was scaled to 0.5 s, equivalent to a heart rate of 120 beats per minute (BPM). It is recognized that this scaling does not account for the changes to the velocity waveform as a result of a different heartrate but is necessary for the data to be compared. Electrocardiogram (ECG) monitoring during the investigation is key to being able to provide the heartrate at the time of the measurement as well as to identify key points in the cardiac cycle such as peak systole and diastole.

In accordance to equation 2, the velocity data at each inlet and outlet patch was multiplied by the respective patch area to calculate the relevant volumetric flow rates which would later be used for boundary condition definition.

(2) $$Q=A_{p\,a\,t\,c\,h}\,v_{a\,v\,e}$$

As the pressure difference is only clinically relevant across the stenosis, the pressure differences measured by Doppler echocardiography at other locations were largely ignored for the purposes of result analysis and boundary condition definitions.

In the post-repair Doppler investigation, the patient was reported to be restless which, with reference to Figure 1, resulted in only measurements G, I, and L being acquired. Assumptions were thus necessary to represent how the inlet flow would be distributed between each arch branch vessel and the descending aorta. Since it was a hypothetical state, case 3 had no data associated with it. Due to the incomplete and absent dataset in case 2 and case 3, respectively, each case required different treatment of volumetric flow plots in order to specify the peak-systolic volumetric flow rate at the boundaries.

**FIGURE 4:**
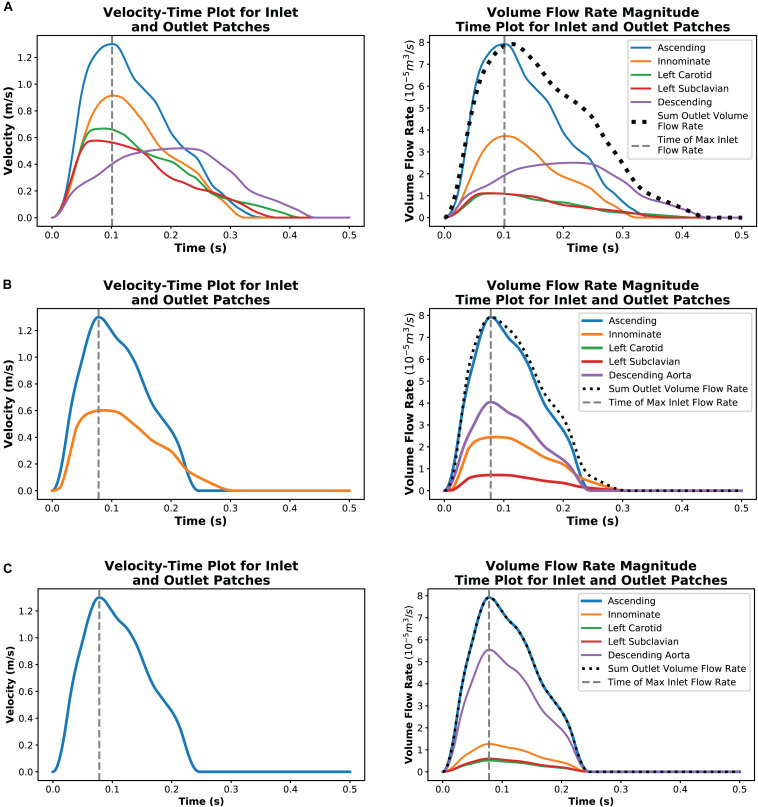
Plots showing a single cardiac cycle of available velocity data for each case and the result of data processing to create volumetric flow rate plots at each outlet for case 1 **(A)**, case 2 **(B)**, and case 3 **(C)**.

The velocity plots derived from the digitized Doppler echocardiography data captures the maximum velocity detected in the region. The volumetric flow rates calculated through equation 2 uses this data and so assumes that the average and maximum velocity are the same. The resulting volumetric flow rate vs. time plots shown in Figure 4 indicate that there was a violation of mass conservation when comparing the sum of the volumetric outlet flow rate and the inlet flow rate. For a rigid wall assumption, this is not the case and so the amplitude of the outlet volumetric flow rates in case 1 were scaled uniformly by a factor of 0.8243 such that the peak value of the sum of the outlet volumetric flow rates matched that of the inlet volumetric flow rate as shown in Figure 4A. This scaling resulted in the least error in the maximum velocity and pressure difference in the region of the stenosis. Other adjustments that were tested and compared included a phase shift of the outlet waveforms such that their peak values (except that of the descending aorta flow) aligned with the peak inlet volumetric flow rate.

In case 2, the innominate artery volumetric flow rate was assumed to have the same scaling factor of 0.8243 as in case 1. It was further assumed that volumetric flow rate ratio in the supra-aortic branch vessels of case 1, relative to the innominate flow, would be consistent in case 2. From the Doppler echocardiography measurements at the site of the stenosis, it is known that there is little to no continuation of flow during diastole through the descending aorta. To ensure that the peak value of the sum of the outlet volumetric flow rates was kept equal to the peak inlet volumetric flow rate, the inlet volumetric flow rate plot was applied to the descending aorta patch but scaled by a factor of 0.5101. Figure 4B illustrates the outcome of this processing method.

The hypothetical case 3 used the ascending aorta volumetric flow rate plot from case 2 as the basis for defining the outlet volumetric flow rates. Studies by Cosentino et al. (2015) and Pirola et al. (2017) indicated that, in healthy aortas, 70–80% of flow may exit through the descending aorta. In case 3, 70% of the flow was set to flow through the descending aorta outlet and the supra-aortic branch vessel flows were divided based on area ratios. The resulting volumetric flow rate plots are illustrated in Figure 4C.

In all cases, the inlet volumetric flow rate was calculated on the assumption that the average velocity was half of the maximum velocity, as for a parabolic profile. The volumetric flow rates of each outlet patch at the point in time where the inlet volumetric flow rate was a maximum were then extracted for the steady-state outlet BC.

The diameter measurement of the coarctation in the post-repair dataset served as geometry data points. In case 2 this was *in lieu* of the CTA data which was not able to be taken post-repair and hence served as a guide for the development of the *in silico* geometry. Due to the lack of PC-MRI data, the implementation of echocardiography proved to be crucial for this study.

### CFD Numerical Modeling

The focus of the study was on the solver and data-processing. Hence it was preferred that a robust and efficient meshing software was used. Thus, each case geometry was meshed in ANSYS ICEM-CFD using tetrahedral cells and five prism boundary layers.

**FIGURE 5:**
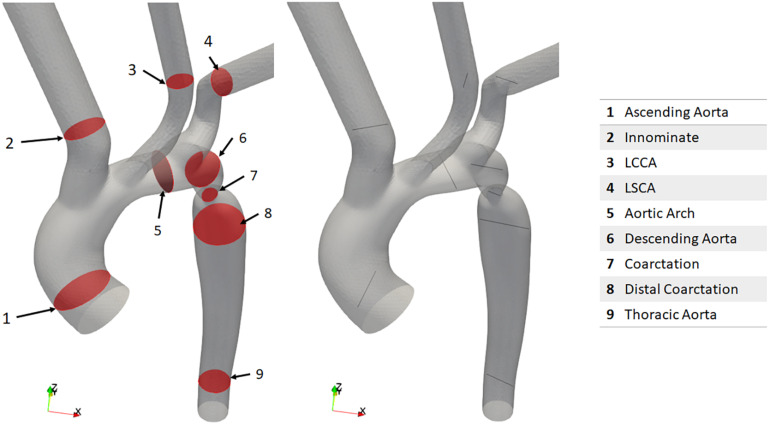
The cross-sections, lines and their naming convention where pressure and velocity were assessed for grid independence.

Through a simplified zero-pressure outlet and peak systolic volumetric flow rate inlet boundary conditions, a grid independence study was conducted for each case using meshes that ranged from *circa* 0.5, 2, and 4 million cells. The metrics that were compared were the average pressures at a cross-section and the velocity profile along a line across the same cross-section. The sample locations are shown in Figure 5 were just distal to the inlet, at each outlet (at the real and not extended location), the aortic arch and proximal, local and distal to the stenosis. It was found that these flow parameters through each geometry were independent within 5% for meshes with approximately two million cells, and as such this resolution was used for the present study.

Steady-state, peak systolic volumetric flow rate boundary conditions were applied at the inlet and outlet patches using OpenFOAM’s built in *flowRateInletVelocity* and *flowRateOutletVelocity* boundary conditions, respectively. These outlet boundary conditions do not impose any set velocity profile at the outlet but rather extrapolate the internal field so that the specified volumetric flow rate is reached. For mass conservation to be satisfied, the inlet volumetric flow rate was set as the sum of the outlet volumetric flow rates in each case. The vessel wall patch was set to have a no-slip boundary condition.

The volumetric flow split assumptions for the outlet boundary conditions required case-by-case assessment. The use of a lumped parameter model, to set boundary conditions based on pre-operative data is preferable as outlined by Morbiducci et al. (2010); Vignon-Clementel et al. (2010). However, in the present study this was not possible due to the lack of clinical pressure and velocity data needed to calibrate the model as discussed in the Methods section. While further approximations could have been made, our aim at this stage was to provide a proof-of-concept study and as such we decided against the introduction of additional uncertainty. Instead, the project focused on directly implementing the Doppler TTE velocity data that was available, while area flow split methods were used so that the pipeline could be established within the means of the clinical infrastructure currently installed. Table 1 collates the volumetric flow rate values that were assigned to each boundary for each case.

**TABLE 1:** Volumetric flow rates applied as outlet boundary conditions for each case.

	Volumetric flow rates $\left(10^{-5}\,\frac{m^{3}}{s}\right)$		
	Case 1	Case 2	Case 3
Inlet	7.84	7.92	7.92
Innominate	3.72	2.94	1.26
LCCA	1.09	0.72	0.51
LSCA	1.09	0.72	0.60
Descending Aorta	1.94	4.04	5.54

Therefore, the Navier-Stokes equations were solved numerically using the open source OpenFOAM (version 6) finite volume method solver. While several open-source solver codes exist, OpenFOAM was selected on account of the breadth of its capabilities and its potential for customization. As the field moves toward greater levels of disease progression and biochemical modeling (Alberto Figueroa et al., 2009), the use of a solver with enhanced capabilities is expected to be important.

The fluid was modeled as an incompressible, laminar Newtonian fluid. The kinematic viscosity was set to 3.78e-6 m^2^.s^–1^ and density to 1060 kg.m^–3^. A higher-order convective spatial discretization and a second order implicit backward Euler temporal discretization scheme were applied (Morbiducci et al., 2010). The pressure field computed by OpenFOAM is the kinematic pressure field and is thus multiplied with density as well as a conversion factor to obtain clinical units of mmHg.

Each case was parallelized using OpenFOAM’s *Scotch* decomposition method. A conservative approach was taken where no more than 50 000 cells were allocated to a CPU core. As a result, 48 CPU cores were used in each case. The Council for Scientific and Industrial Research’s (CSIR) High Performance Computing (HPC) cluster located in Cape Town, South Africa was used and due to the use of OpenFOAM, there was no cost-implication.

The computational time for case 1, case 2 and case 3 were approximately 17, 8.5 and 8.5 hours, respectively. The solution for case 2 and case 3 were found to be significantly faster due to the generally lower velocities present in the stenosed region, which allowed for larger time steps to be taken.

## Results

### Geometry Segmentation and *in silico* Design

**FIGURE 6:**
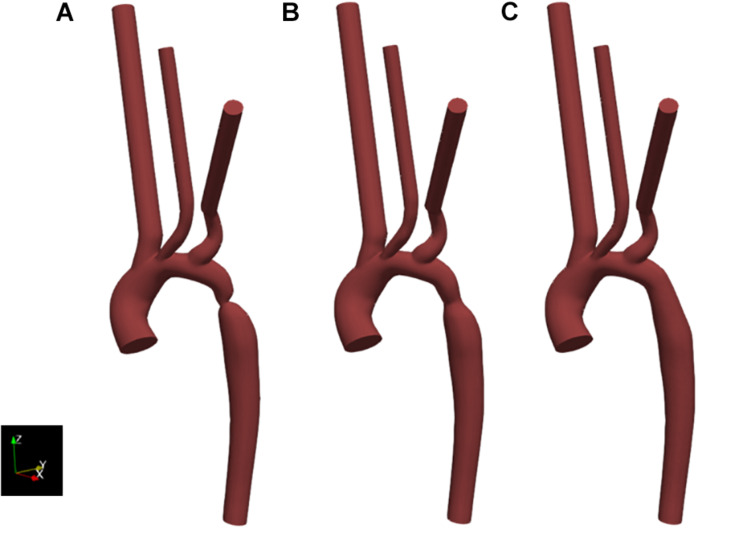
Resulting geometries of segmentation and subsequent adaptation to represent the geometry of the pre-intervention state (case 1) as extracted from CT data **(A)**, as well as an approximation of the post-intervention state (case 2) **(B)** and a healthy or totally repaired aorta (case 3) **(C)**. Geometries include the extended outlets which were artificially generated for numerical stability.

The segmentation of the pre-repair, and the design of the post-repair and healthy geometry cases are shown in Figure 6.

The coarctation ratios were defined as the ratio between the minimum diameter in the coarctation region and the diameter of the descending aorta at the level of the diaphragm and is shown in Table 2 for each case as well as supporting data. These ratios indicate the severity of the coarctation in the region and, although there is significant improvement post-repair, there still existed a small stenosis.

**TABLE 2:** Coarctation ratios calculated for each repair case by the standard set by Forbes et al. (2011).

Geometry	Coarctation Area (mm^2^)	Perimeter (mm)	Hydraulic Diameter (mm)	*D*_*Coarct*_: *D*_*DAo*_
Case 1	8.08	9.59	3.37	0.43
Case 2	25.91	17.99	5.76	0.74
Case 3	61.82	27.61	8.96	1.14
Descending Aorta	48.25	24.66	7.83	–

### Doppler Echocardiography Data Collection and Processing

An example of the Doppler echocardiography data at the ascending aorta is shown in Figure 3 where a single cardiac cycle from the pre- and post-repair intervention is highlighted for digitization for use in the model’s boundary conditions. Here it can be seen that there are variations in the shape of the velocity-time profile as well as the clarity of the echocardiography measurement. The Doppler echocardiography data that was collected pre- and post-repair are shown in Table 3.

**TABLE 3:** Collation of data that was acquired from the Doppler transthoracic echocardiographic imaging as a part of the data collection protocol developed for this study.

Location	Max velocity (m/s)	Max pressure difference (mmHg)	Measured Diameter (mm)
**CASE 1**			
Ascending aorta	1.3	6.80	13.00
Innominate	1.11	4.93	8.15
LCCA	0.81	2.60	4.30
LSCA	0.70	1.95	5.00
Coarctation	3.49	48.65	–
Descending aorta	0.63	1.59	8.29
**CASE 2**			
Ascending aorta	1.3	6.76	11.21
Innominate	0.84	2.82	6.98
Coarctation	2.38	22.72	6.00
LCCA	–	–	–
LSCA	–	–	–
Coarctation	–	–	–
Descending aorta	–	–	–

*It should be noted that, although the maximum pressure difference at each location was recorded through the Doppler echocardiography, there was only interest in the measurement across the coarctation due to its significance for the assessment of coarctation of the aorta.*

Table 3 includes the maximum velocity in the cardiac cycle, the maximum pressure difference in the cardiac cycle calculated through the simplified Bernoulli equation in equation 1 and the diameter of the vessel where the measurement was taken. It is important to note that measured diameters at the ascending aorta and innominate arteries in cases 1 and 2 differ by more than 10%, which is attributable to differences in spatial alignment of the measurements made in each case. For this reason, only the diameter at the coarctation site was fed back into the geometry design in case 2.

### Velocity Results

**FIGURE 7:**
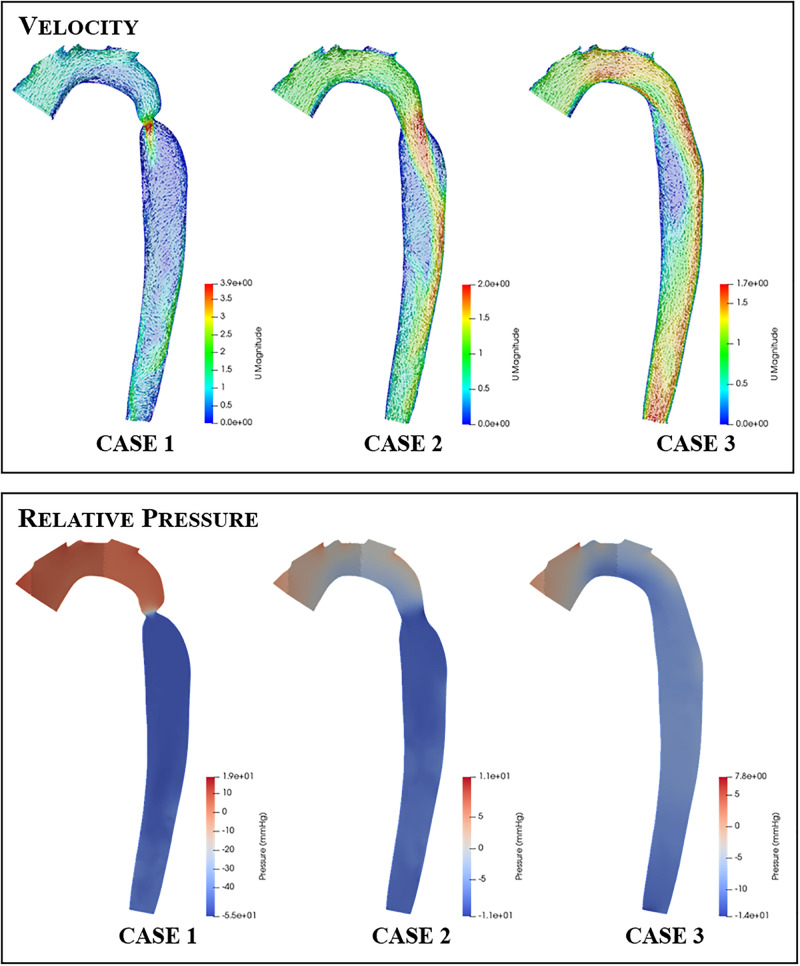
Contour and vector plots along a slice through the aortic arch and descending aorta of the velocity (top row) and pressure (bottom row) fields for each repair case.

**FIGURE 8:**
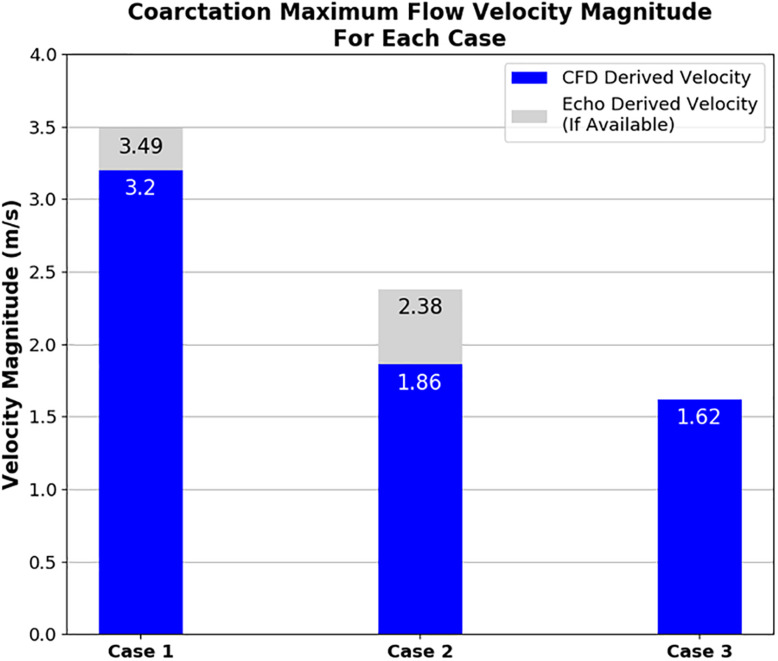
Plot showing the maximum velocity through the coarctation derived from CFD in comparison to echocardiography measurements for each case of coarctation repair. Note that echocardiography data was not available for case 3.

Figure 7 clearly shows that, in cases 1 and 2, the velocity magnitude reaches a maximum in the vicinity of the coarctation constriction as expected. In addition, Figure 8 shows that the magnitude of these peak velocities in the region of the coarctation reduces from 3.24 m.s^−1^ in case 1 to 1.62 m.s^−1^ in case 3. Considering the echocardiography data, the maximum coarctation velocity of 1.86 m.s^−1^ in case 2 is found to be 22% lower than the Doppler echocardiography measurement of 2.38 m.s^−1^.

The qualitative impact of the intervention on the flow field distribution is also apparent in the aortic slice shown for each case in Figure 7. The high flow velocities through the coarctation in case 1 is seen to create large recirculation zones, particularly in the region of post-stenotic aortic dilation. Furthermore, the flow in the rest of the descending aorta shows evidence of helical flows and in general the flow in this region does not recover to a uniform axisymmetric state. In contrast, the fluid flow in cases 2 and 3 exhibit smaller recirculation zones, more ordered flow and better developed flow at the outlet of the descending aorta. The recirculation zone on the right-hand side wall, distal to the coarctation zone, reduces and disappears as the coarctation is expanded in each case.

Table 4 adds further qualitative data to illustrate the impact of the coarctation expansion by presenting detail of the velocity magnitude contours in the supra-aortic branch vessels and descending aorta. It was found that there was considerable change to the contours as the repair progressed from case 1 to case 3. Furthermore, the velocity magnitudes in each supra-aortic arch vessel showed evidence of reducing as the coarctation was expanded. Conversely, the flow through the thoracic aorta increases drastically as the coarctation expands and tends toward a more consistent profile. It is easy to see through this visualization that the flow is redistributed from the supra-aortic branch vessels to the descending aorta, as the coarctation is expanded. Granted, the boundary conditions enforce such a trend, but this data may further present a potential value to procedural planning where enhanced hemodynamic detail can be provided to clinicians.

**TABLE 4:** A qualitative comparison of the velocity magnitude contours between the different repair cases at each major outlet.

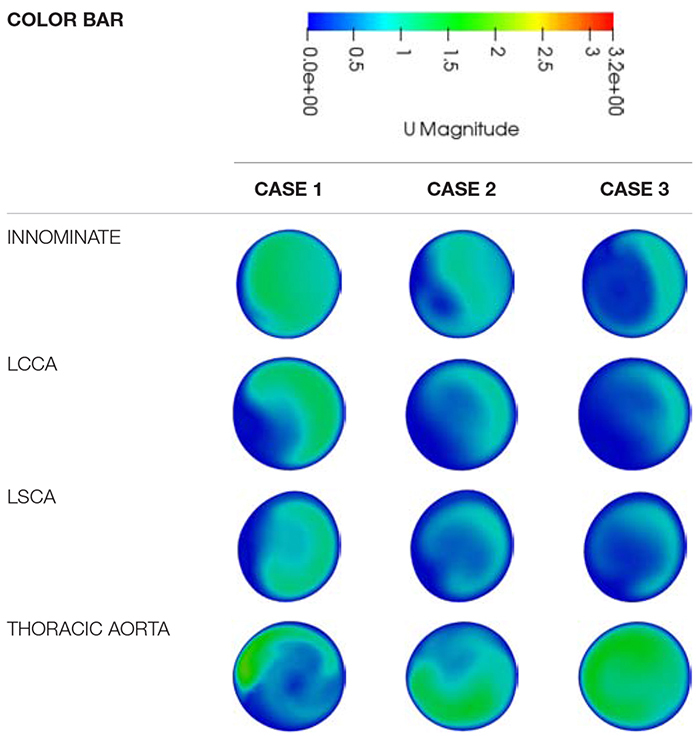

*Location labels relate to Figure 5.*

### Pressure Results

**FIGURE 9:**
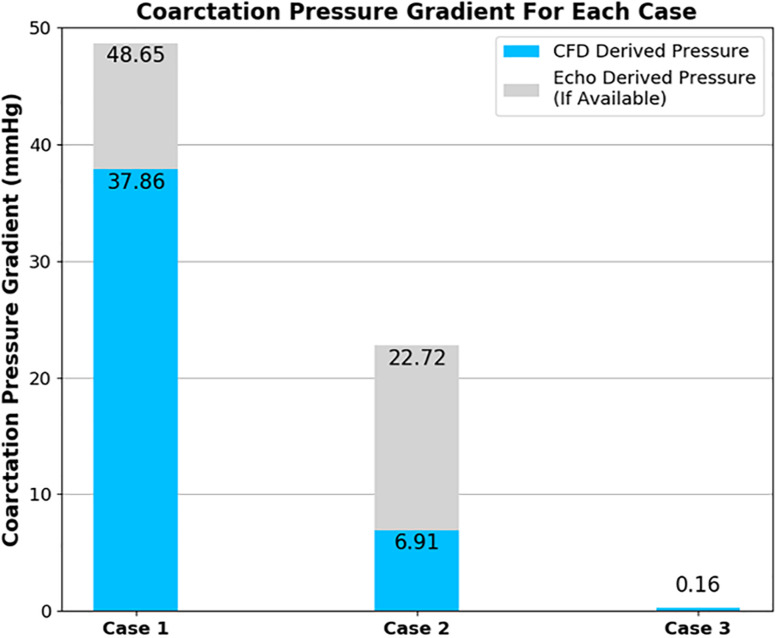
Plot showing the pressure difference across the coarctation derived from CFD in comparison to echocardiography measurements for each case of coarctation repair. Note that echocardiography data was not available for case 3.

The pressure difference across the coarctation is an important metric since it is so closely tied to the clinical assessment of a CoA patient, and the subsequent repair decision. The CFD calculated pressure differences are shown in Figure 9. In Figure 7, it is important to note that the pressure field displayed is a gauge pressure rather than an absolute value. Since the focus of this study is on the pressure difference across the stenosis rather than the absolute pressure at a given point, this was deemed to be sufficient.

The CFD prediction for case 2 indicates a reduction of peak velocity through the coarctation and a corresponding reduction in the size of the recirculation zone and downstream flow disturbance in the descending aorta. Despite this qualitative improvement, Doppler echocardiography still recorded a pressure difference in excess of the 20-mmHg standard for indicating the need for intervention. The pressure differences are collated in Figure 9 and indicate that, in case 1, both the measured and the predicted gradient is in excess of the 20-mmHg standard, indicating the presence of a stenosis that requires repair. The measured pressure difference was somewhat higher than the predicted value, which is in part anticipated, due to the use of the simplified Bernoulli equation in the echo-derived measurement (Singh et al., 2019), which implies no flow separation. As observed from the predicted flow field in Figure 7, this is a significant assumption. In case 2, the predicted pressure difference is again lower than the measured value, although in this case by a greater margin. However, the approximations made to boundary condition flow rates for this case are expected to be contributing to this discrepancy.

## Discussion

### Feasibility of Patient Data Acquisition

The results of the process of obtaining and assessing the echocardiography data served two purposes. Primarily, this data would be used for defining the boundary conditions for the CFD simulation of flow in each case. In parallel to this, the collaboration with clinicians to develop the data collection protocol, obtain the data and assess its quality allows for the evaluation of the advantages and disadvantages of using echocardiography as a modality for data collection. The pre-intervention echocardiography data in case 1 showed that echocardiography was capable of obtaining flow and geometry data to be used in defining the boundary conditions at each inlet and outlet patches of an aorta model.

Echocardiography has important advantages which make it attractive as a modality for patient-specific CFD data collection protocols, as was experienced in this study. Apart from its high spatial and temporal resolution, the most important advantage that echocardiography holds over CTA and MRI is the ease with which it can be applied, and the low risk associated in doing so. This was a particularly important ethical factor in implementing the protocol as it would be necessary to repeat investigations at various points in time.

On a more pragmatic note, an MRI facility was not available at Red Cross for the patient in this study. As such, the use of echocardiography to provide velocity information is indispensable in the case where the use of CTA and/or MRI is limited due to safety, ethical or logistical reasons.

The quality of echocardiography is known to be dependent on patient cooperation, as well as the skill level of the operator. One only needs to consider the contrast in success of collecting a full dataset shown in Table 3 from the pre-intervention to the post-intervention cases to see how the acquired data can vary from one investigation to the next. In a broader implementation of this protocol, it should be expected that since different members of staff would be carrying out the investigation, this adds a further variable to the data collection. Hence, it would be appropriate that the most experienced operator should be involved in data acquisition particularly when developing CFD studies for use as a clinical tool.

When relying on Doppler echocardiography as the sole modality for obtaining boundary condition data, there is likely to be a need for a higher degree of data pre-processing than shown in current literature for PC-MRI derived boundary conditions (Pirola et al., 2017). Aside from the difference in processing the clinical data to convert velocity data into volume flow rate, 2D color Doppler scans, unlike PC-MRI, can only show the magnitude of the velocity in a single plane. However, despite the challenges of implementing echocardiography, it should not be abandoned as a modality for data collection in CFD studies. Rather, due to it being a modality that is heavily relied on in many LLMICs, these challenges should be addressed by the development of methods that appropriately account for these uncertainties.

### Hemodynamics

The improvement of flow features observed qualitatively in Figure 7 and Table 4 demonstrates the benefit of CFD as a tool for clinicians to visualize the flow and pressure fields as a result of a specific repair, prior to the procedure. It is known that unfavorable hemodynamics can drive the development of atherosclerosis (Cecchi et al., 2011) or persistent hypertension (Heath et al., 1986), for example. Thus, computational modeling of a repair procedure offers visualization and quantification of the changes to the velocity and pressure fields that may highlight risk factors for developing such morbidities and others.

With the data that was available, the methodology recreated the peak velocity magnitude value through the coarctation zone within 10% accuracy in case 1. While a high peak velocity value may be indicative of a jet caused by a stenosis, it is not necessarily the principal diagnostic metric used by clinicians. However, in considering the data that could be collected within the bounds of what was feasible at Red Cross, this peak velocity was helpful in a workable level of agreement with the CFD. Agreement between the CFD and Doppler echocardiography stenosis velocity measurements was especially important from an engineering perspective, due to the relationship between the velocity of blood flow through the stenosis and the pressure difference across it which is one of the focal points of this research.

The method used to calculate the outlet volumetric flow rates for case 2 resulted in the maximum velocity at the coarctation zone being close, albeit lower, to the echocardiography measurement. Despite this, the CFD pressure measurement was lower than the echocardiography measurement. Compared to the 20-mmHg threshold for intervention, the difference between the CFD and Doppler echocardiography measured difference changes the indicated outcome of the repair where the CFD result indicates an adequate repair as opposed to the Doppler echocardiography measurement which, in isolation, would not.

Where CFD measurements are being used for decision-making (be it in diagnosis or repair planning), it is important to consider whether these results would otherwise impact the decision. This is particularly important where the CFD results give a false indication of severity and, consequently, the patient is not treated appropriately. In this regard, it’s important to note that a clinical assessment of the severity of CoA depends on several measurements.

In the post-repair Doppler echocardiography investigation of the patient in this study, there was supporting evidence of a successful repair particularly due to the reduction of the diastolic tail. This refers to the continuation of flow through the stenosis during diastole due to the distensibility of the aortic arch and is directly proportional to the severity of the stenosis. Other metrics which guide clinical decision making may include normal blood pressure, presence of palpable distal pulses and normal left ventricular function and hence the echocardiography pressure difference measurement was not taken into account.

Provided that a computational model is validated and adequately defined, the pressure difference predicted by a CFD study would be more reliable, given that it is calculated from the comprehensive Navier-Stokes equations as opposed to the comparatively simple form of the Bernoulli equation used in Doppler echocardiography, which are known to overestimate the pressure difference in such flows (Goubergrits et al., 2019).

Having a more reliable non-invasive pressure difference measurement would be a key benefit of applying CFD in a clinical setting. In addition, computational results and their analysis can be conducted at any point in the domain with a better consistency and higher resolution than Doppler echocardiography. This provides greater freedom for clinicians to analyze patient hemodynamics as it would remove the typical restrictions otherwise caused by acoustic windows, patient restlessness or the angle that the echocardiography probe makes with the blood flow.

While CFD can add another modality into the set of diagnostic metrics available for clinical decision making, another key role for a well-validated CFD approach lies in the *in silico* modeling of blood flow for given repairs to add foresight during repair planning.

The implication of these results is that, the pressure difference has been reduced to a safe level without the need to expand the aorta as far as was modeled in case 3. In the case of a balloon dilation procedure, this avoids expanding the vessel to a point where there is a significant risk of damage to the vessel. However, the resulting elevated residual pressure in the aortic arch in case 2 would be beneficial information for assessing the risk of the patient developing other morbidities later in life and, consequently, planning the management strategy to do one’s best to mitigate this.

These kinds of insights are in fact the general potential that is described in much of cardiovascular CFD literature, however, it is important to highlight that, in many cases and indeed in this study itself, validation of results is highlighted as being crucial for the safe implementation of any tool and prior to these derived results being used for decision making (Cosentino et al., 2015; Marsden and Feinstein, 2015; Rinaudo et al., 2015).

## Conclusion

This study presents a patient-specific CFD pipeline for the study of coarctation of the aorta which is specifically feasible for clinical application within a resource-constrained environment. The data collection protocol is emphasized due to its use of Doppler echocardiography as a modality for collecting velocity flow data. The capability of PC-MRI is not contested, but, Doppler echocardiography is argued to be an important tool to be able to implement CFD studies where advanced facilities such as PC-MRI do not exist.

The velocity and pressure data which resulted from these studies show how patient-specific computational modeling could provide complimentary diagnostic insight for more rigorous assessment of CoA and present hemodynamic information for the optimization of *in silico* repairs while being feasible within less resourced healthcare facilities.

## Data Availability Statement

The datasets generated for this study are available on request to the corresponding author.

## Ethics Statement

The studies used secondary data from the Red Cross War Memorial Children’s Hospital. Patient consent was obtained and data was anonymised prior to its use. The study was reviewed and approved by the University of Cape Town Ethics Committee HREC Ref R017/2014.

## Author Contributions

LS contributed to the design of the protocol and methodology, analysis, and writing of the manuscript. MN, LZ, AR, BO, AK, and AD contributed to the conceptualization of the work, analysis, methodology development, and critical revision of the manuscript. TA, JL, RD, BF, and GC contributed through protocol and methodology development, data collection and result analysis, and verification. LZ, BK and ME contributed through conceptualization and fiscal support. All authors contributed to the critical revision of the manuscript and agreed to be accountable for all aspects of the work.

## Conflict of Interest

The authors declare that the research was conducted in the absence of any commercial or financial relationships that could be construed as a potential conflict of interest.
